# Fast and Automated Segmentation for the Three-Directional Multi-Slice Cine Myocardial Velocity Mapping

**DOI:** 10.3390/diagnostics11020346

**Published:** 2021-02-19

**Authors:** Yinzhe Wu, Suzan Hatipoglu, Diego Alonso-Álvarez, Peter Gatehouse, Binghuan Li, Yikai Gao, David Firmin, Jennifer Keegan, Guang Yang

**Affiliations:** 1 National Heart & Lung Institute, Faculty of Medicine, Imperial College London, London SW7 2AZ, UK; p.gatehouse@rbht.nhs.uk (P.G.); d.firmin@imperial.ac.uk (D.F.); j.keegan@rbht.nhs.uk (J.K.); 2 Department of Bioengineering, Faculty of Engineering, Imperial College London, London SW7 2AZ, UK; binghuan.li19@imperial.ac.uk; 3 Cardiovascular Biomedical Research Unit, Royal Brompton Hospital, London SW3 6NP, UK; s.hatipoglu@rbht.nhs.uk; 4 Research Computing Service, Information & Communication Technologies, Imperial College London, London SW7 2AZ, UK; d.alonso-alvarez@imperial.ac.uk; 5 Department of Computing, Faculty of Engineering, Imperial College London, London SW7 2AZ, UK; yikai.gao18@imperial.ac.uk

**Keywords:** cardiovascular, segmentation, deep learning

## Abstract

Three-directional cine multi-slice left ventricular myocardial velocity mapping (3Dir MVM) is a cardiac magnetic resonance (CMR) technique that allows the assessment of cardiac motion in three orthogonal directions. Accurate and reproducible delineation of the myocardium is crucial for accurate analysis of peak systolic and diastolic myocardial velocities. In addition to the conventionally available magnitude CMR data, 3Dir MVM also provides three orthogonal phase velocity mapping datasets, which are used to generate velocity maps. These velocity maps may also be used to facilitate and improve the myocardial delineation. Based on the success of deep learning in medical image processing, we propose a novel fast and automated framework that improves the standard U-Net-based methods on these CMR multi-channel data (magnitude and phase velocity mapping) by cross-channel fusion with an attention module and the shape information-based post-processing to achieve accurate delineation of both epicardial and endocardial contours. To evaluate the results, we employ the widely used Dice Scores and the quantification of myocardial longitudinal peak velocities. Our proposed network trained with multi-channel data shows superior performance compared to standard U-Net-based networks trained on single-channel data. The obtained results are promising and provide compelling evidence for the design and application of our multi-channel image analysis of the 3Dir MVM CMR data.

## 1. Introduction

Three-directional cine multi-slice left ventricular myocardial velocity mapping (3Dir MVM) is a promising cardiac magnetic resonance (CMR) technique to assess cardiovascular conditions. In addition to conventionally available magnitude data, this technique also acquires velocity-encoded phase velocity mapping data from the phase-contrast MRI without ionizing radiation [1,2,3]. These phase velocity mapping data are used to generate velocity maps in three orthogonal directions throughout the cardiac cycle to gather both spatial and temporal information from the cardiac movement. The cine data also provided us with an opportunity to observe the cardiac movements and segment the left ventricular myocardium by employing the available temporal data. 

Delineating the left ventricle (LV) myocardium from the CMR data is crucial in the determination of the myocardial motion from this data. Manual segmentation of both endocardial and epicardial borders of the LV myocardium is a very tedious process taking up to an hour for an experienced clinician to complete a single-slice 50-frame study. There can also be subjectiveness and variations in the manually segmented myocardium when the myocardium of the same subject is segmented when repeated over time or by another experienced clinician.

Following successful applications of deep convoluted neural networks (CNNs) [4], in particular the U-Net [5], for natural image [6] and medical image processing [7,8,9] for the brain [10,11,12], liver [13], breast [14], lung [15,16] and other regions [17], we here assess and prove that the U-Net-based model can be applied to 3Dir MVM CMR single-channel phase velocity map images and single-channel magnitude images. Furthermore, motivated by the growing interests in medical image fusion [18], especially in multi-channel processing in biomedical imaging and architectural modifications to encoder-decoder to improve the performance of neural networks [19], we advance our method to encode both magnitude and phase velocity mapping data from 3Dir MVM CMR with the implementation of attention modules, so the model can observe these multi-channel images with focuses. Moreover, the traditional strategy assumes simple (e.g., linear) relationships between channels, which may not represent the reality correctly [20]. Therefore, we propose a model to incorporate dense connections on all layers of depth of CNNs instead of the traditional single-layer fusion to model the relationships among all channels. This model is able to load and infer on each cine slice within less than 15 seconds using a graphics processing unit (GPU) specified in Section 2. 

In evaluating LV contours (i.e., the LV epicardial and endocardial contours) generated against the manually delineated ground truth (initialized via an active contour segmentation), we employ both the widely used method of Dice Scores and our novel comparison methodology using the quantitative myocardium CMR velocity mapping (MVM) [21,22] data generated. By employing both metrics, we can observe the accuracy of the segmentation by not only overlaps of each image array but also the time derivatives of the movements of the segmented myocardium, i.e., their velocities, within an electrocardiogram (ECG) R-R cardiac cycle by taking advantage of the cine MRI acquired. By evaluating model generated segmentation based on the accuracy of the CMR MVM results, we can observe and further compare the segmentation performance on clinically important markers derived from these velocity curves. 

Despite the remarkable performance of existing methods in LV myocardial segmentation of CMR single-channel magnitude images, the combination of multi-channel data of 3Dir MVM CMR at various levels of abstraction has not been exploited. Our work addresses this problem by assessing, investigating and comparing different strategies of incorporating and analysing CMR multi-channel data using both Dice Scores and clinically relevant CMR MVM results.

Additionally, we also invited a second human observer to conduct an inter-observer study between the second and the first observers and between the model and the first observer, to see the human inter-observer variations and reproducibility compared to the model vs. human operator performance on CMR MVM. 

To summarize, the novelties of this work are of 3-fold: (1) we assessed and proved that U-Net can be applied to single-channel magnitude data, single-channel phase velocity mapping data and their combination, as multi-channel data, respectively, from 3Dir MVM CMR for automated LV myocardium segmentation; (2) we designed and proposed a novel multi-channel attention block (MCAB) and a novel segmentation framework to better incorporate and segment the multi-channel data from 3Dir MVM CMR; (3) we evaluated the clinical accuracy of the novel segmentation framework by comparing outputs with the myocardial velocities and widely clinically applied markers obtained from manual segmentation, to assess the segmentation results from the LV myocardium velocity curves and markers by taking advantage of the cine MRI acquired. In addition, we further compared the variation and reproducibility of the segmentation between the model and a second clinical human observer from the first observer. We also provided a reference of human inter-observer variations in the myocardium segmentation in terms of their Dice Scores.

## 2. Materials and Methods 

### 2.1. Data Acquisition and Pre-Processing

The CMR data were acquired from 18 healthy subjects (8 of them were acquired twice, giving 26 datasets in total) at the Royal Brompton Hospital. There was an additional subject acquired twice for independent testing (Section 3.2). All 19 subjects (18 for training (26 datasets) and 1 for independent testing (2 datasets)) had normal sinus rhythms. Further demographic information of these 19 subjects is included in Table 1.

For each subject, high temporal resolution (reconstructed to 50 frames per cardiac cycle, i.e., per R-R interval in the electrocardiogram (ECG)) cine spiral MVM with non-Cartesian SENSE reconstruction and 3 orthogonal directions of velocity encoding were acquired in a single breath-hold [21]. There were 121 cine slices (giving 6050 cine frames in total) in total from all these datasets (3–5 cine slices for each dataset). The acquired spatial resolution was 1.7 × 1.7 mm^2^, and data were reconstructed into a 512 × 512 matrix (reconstructed spatial resolution 0.85 × 0.85 mm^2^). Data were acquired in short-axis slices from base to apex of the left ventricle. 

All subjects gave their informed consent for inclusion before they participated in the study with approval from the local institutional review board (in accordance with the Declaration of Helsinki). The institutional review board approved the CMR scanning (Development of Cardiovascular Magnetic Resonance Sequences for Future Clinical Use V3.0 October 10, 2018, approved by the NRES London East Research Ethics Committee). The ground truth of the myocardium segmentation was performed by a senior CMR physicist with 20+ years’ experience in CMR. 

We augmented the data by a random horizontal flipping (probability of 0.5) and a random rotation (angle = [0, 90°]) before the training.

### 2.2. Network Architectures for the Myocardium Segmentation

We summarized the available standard strategies on supervised learning-based deep learning as below, where they are all U-Net-based approaches.

[a] U-Net with CMR single-channel magnitude data (512 × 512 × 1), abbreviated as U_M below;

[b] U-Net with CMR single-channel phase velocity mapping data (512 × 512 × 3), abbreviated as U_P below;

[c] U-Net with CMR multi-channel data, where we stacked the magnitude (512 × 512 × 1) and phase velocity mapping (512 × 512 × 3) data on the channel axis of the input image, abbreviated as U_MP below.

In addition, we designed the strategies below, allowing the model to understand the more latent relationships between different channels.

**Figure 1:**
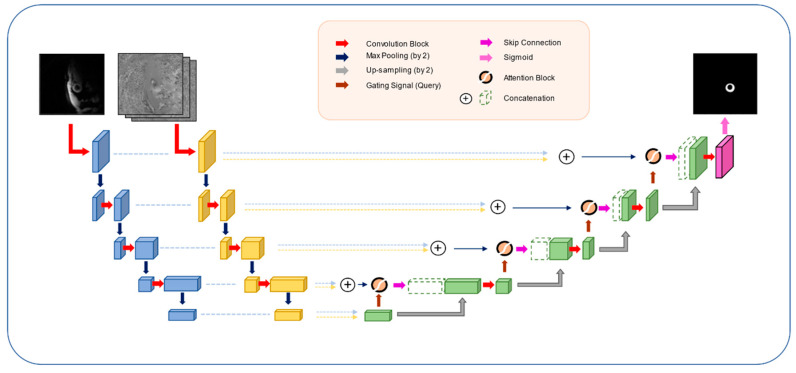
Network architecture of our proposed myocardium segmentation in three-directional cine multi-slice left ventricular myocardial velocity mapping (3Dir MVM) cardiac magnetic resonance (CMR) (i.e., model [d] in Section 2.2).

[d] We proposed a novel network structure (Figure 1) based on standard U-Net structures and its encoders and decoders, where we input the magnitude (512 × 512 × 1) and phase velocity mapping (512 × 512 × 3) data separately into the network. Each input had its respective encoder to allow the model to extract information that would otherwise be fused at early stages. We then added a novel MCAB to disentangle the multi-channel information better before it entered the decoder following standard U-Net structure at each depth of the network. This model is abbreviated as AMU_MP below.

Within MCAB, we firstly concatenated the outputs of two encoders at each depth and added a convolutional block that was of the same setting as the encoder to allow the model to disentangle the latent relationship between the information between the two channels. Then, the convoluted data stream went through an attention block [23] before being delivered to the decoder at each depth.

### 2.3. Training Procedure

For the 121 CMR cine slices (each of 50 frames, giving 6050 2D cine frames in total) introduced in Section 2.1, we augmented each cine slice 4 times. Then, we stacked all augmented cine CMR cine frames and the original cine CMR cine frames on their frame axis, giving a total of 30250 stacked frames (6050 × 4 + 6050 = 30250) for training. All models specified in Section 2.2 were trained from scratch and evaluated using a five-fold cross-validation scheme. As 121 is not a multiple of 5, each validation round takes 23–25 original CMR cine slices (1150, 1200 or 1250 testing frames) for testing and the augmented and original cine frames corresponding to the rest of the cine slices for training. We trained our network with cross-entropy loss using the Adam optimizer [24]. For hyperparameters per Kingma et al. [24] we set α = 0.001, β_1_ = 0.9, β_2_ = 0.999, ε = 1× 10^−7^.

For all experiments, we defined the batch size as 8 to accommodate the high image resolution of 512 × 512. The model was trained for 5 epochs. The training was completed with an NVIDIA TESLA V100 GPU.

### 2.4. Testing Procedure

The input for testing was prepared in the same way as training as part of the five-fold cross-validation. The testing was firstly performed on an NVIDIA TESLA V100 GPU and then repeated on a machine without a GPU to test its efficiency on a modern computer without a powerful GPU. This can also prove the feasibility of our model to be deployed in a clinical environment, in which normally a decent GPU may not be available.

### 2.5. Post-Processing

For healthy subjects, the endocardial and epicardial borders of their CMR short-axis data were expected to be approximately ellipsoid. Any “broken” segmentations, which did not conform to this shape constraint, passed through an additional postprocessing stage. Examples of “broken” segmentation included semi-ellipses and ellipses that were not closed. 

**Figure 2:**
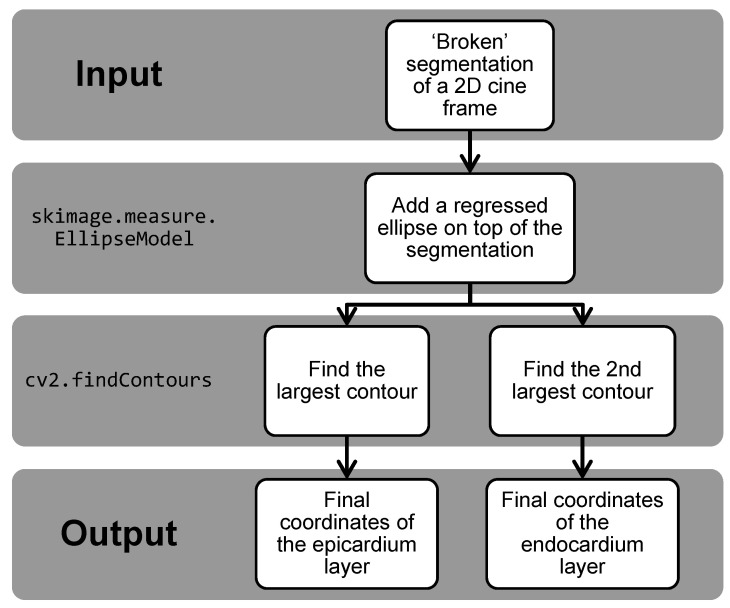
Flow chart of the post-processing workflow.

At the post-processing stage, we followed Halir et al. [25] to regress the predicted segmentation around the centre of the segmentation into a 3-pixel width ellipse, and we output the final prediction as the regressed ellipse on top of the originally predicted segmentation so that any “broken” segmentation could be rectified. Then, the largest and the second largest contours obtained were considered as the final coordinates of the epicardium and the endocardium layer, respectively, for the generation of CMR MVM. A flow chart is included (Figure 2) for a clearer explanation of the workflow. 

In addition, the analysis of the myocardial velocities in Section 2.6.2 requires the segmentation results generated to be a close ring-like shape, so it can calculate the velocity of movement for the whole myocardium. Therefore, this post-processing also helped to shape the model-generated results to be an acceptable input into the myocardium velocity analysis tool, so we can analyse these velocity data and markers as described.

### 2.6. Evaluation Metrics

#### 2.6.1. Dice Similarity Coefficient (or Dice Scores)

Dice Similarity Coefficient (DSC) is a broadly used metric to evaluate the segmentation performance based on the overlap between the automated segmentation result and the ground truth [26,27]. Let *S_auto_* and *S_gt_* be the automated segmentation result and the manually annotated ground truth, respectively; the DSC can be defined as the Equation (1) below [26,27].

(1) $$\mathrm{DSC}\left(S_{auto},S_{gt}\right)=\frac{2|S{\;}_{auto}\cap S_{gt}|}{|S_{auto}|\;+\;|S_{gt}|}.$$

To assess and compare the performance of each model, we first evaluated the accuracy of predictions using Dice Scores on raw predicted segmentation data before post-processing (1) per cine frame, (2) per cine slice (50 cine frames) and (3) per subject (3–5 cine slices). The three Dice Score metrics allowed a comprehensive observation and examination of the model’s performance.

#### 2.6.2. CMR MVM Velocity Data

While the Dice similarity coefficients examine the overlaps per segmentation data array, the CMR MVM velocity analysis observes the temporal derivatives of the 3D movements of the segmented LV myocardium, i.e., velocities, by three axes—the longitudinal, radial and circumferential [21].

The LV myocardial velocity analysis, especially regionally, has the potential to be recognised under clinical settings to analyse the function of the myocardium in terms of its temporal movements (i.e., LV contraction and relaxation).

After segmentation, global longitudinal, radial and circumferential LV myocardial velocities were calculated and recorded from simulated temporal LV myocardial movements within a cardiac cycle according to the LV myocardial segmentations. By analysing the curves, global peak radial/longitudinal velocities were recorded for systolic (PS), diastolic (PD) and atrial systolic (PAS) velocities at all slices across the axial direction. Additionally, from the circumferential velocity–time curves, we recorded the typically shown two early circumferential systolic peaks (C1 and C2) and a third circumferential early diastolic peak (C3). All these peaks are widely applied as markers in the myocardial movements under clinical settings and in reproducibility and reliability analysis [28,29]. In addition to the global velocity data, we split the myocardium into 6 regions in accordance with the advice from the American Heart Association [30]—anterior (A), mid anteroseptal (AS), inferoseptal (IS), inferior (I), inferolateral (IL) and anterolateral (AL), and reported all peak velocities for each region as well. From these peak velocity data collected, we can see how these variations may be able to help to form clinically valuable evidence for diagnosis.

In this study, we were able to evaluate these velocity curves and markers for a total of 8 subjects, each of whom was scanned twice, giving 16 datasets. We were not able to analyse the rest of subject population, since these datasets were acquired and processed by another system, which was incompatible with our current CMR MVM myocardium velocity analysis tool. However, the analysed 16 datasets are representative.

To assess the clinical significance of the predicted segmentation and its accuracy for obtaining clinical measurements, we compared the longitudinal, radial and circumferential markers of peak velocities from the LV myocardial velocity curves generated from the manual segmentation and the model-generated delineation using two-way mixed, single score inter-class coefficients (ICCs) [31,32,33] and also the Bland–Altman analysis. For ICC, we classified them as excellent (>0.75), good (0.6 < ICC ≤ 0.75), fair (0.4 < ICC ≤ 0.6) and poor (<0.4) per each region for regional peak values and per whole myocardium for global peak values [31].

### 2.7. Inter-Observer Study

We then invited another very skilled and experienced clinician (10+ years in CMR) as our second observer to delineate two randomly picked datasets independent of the first observer who previously defined our ground truth. 

We compared the Dice Score distributions between the first observer and the second observer and the Dice Score distributions between the first observer and our best performing model (model [d] as defined in 2.2) in terms of their mean Dice Scores. 

The model employed for testing here refers to one of the five cross cross-validation trained models. This model is trained by the corresponding augmented cine frames and original cine frames of 97 CMR cine slices. 

This comparison can help us to see the variability of segmentation results of the model and the second human observer compared to the first human observer in terms of their segmentation overlaps by Dice Scores.

## 3. Results

### 3.1. Cross-Validation Testing

We cross-validated all four models (Section 2.2 [a]–[d]) to demonstrate their capabilities in CMR MVM myocardium segmentation using the two evaluation methodologies. Then, we compared the results of different models by the mean and standard deviation. Results were tested for statistically significant differences using the Wilcoxon signed-rank test, and the tests were considered significant if associated *p* values were <0.05.

**Figure 3:**
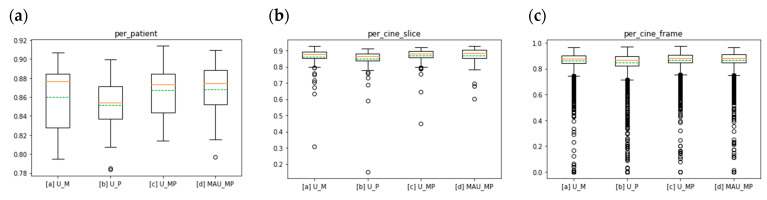
Boxplot of Dice Scores for different models (**a**–**c**), and [d] per subject, (**a**) per cine slice (**b**) and per cine frame (**c**) of the comparison study results in five-fold cross-validations using different methods. In the boxplot, the orange line indicates the median value, the green line indicates the mean value and the dots around indicate the outliers. Outliers here are defined as data points above Q3 + 1.5 × IQR or below Q1 − 1.5 × IQR of the distribution. (Q1: first quartile, Q3: third quartile, IQR: interquartile range (Q3–Q1).)

The quantitative results of the models validated by Dice Scores can be found in Table 2 and Figure 3. As the differences between models are relatively small, we conducted statistical tests to evaluate the differences more critically. By comparing the results between single-channel input-based models in Section 2.2 [a] and [b] and the multi-channel input-based models in Section 2.2 [c] and [d], we can see that the multi-channel models [c] and [d] yielded higher Dice Scores, where model [d] resulted in the highest in particular. The performance of models [c] and [d] is confirmed by the statistical significance (*p* < 0.05) when comparing the per cine frame Dice Scores of [c] and [d] against the results of [a] and [b] (Table 3c). Our proposed model [d] yielded the highest mean Dice Scores, confirmed by the statistical significance (*p* < 0.05) when comparing the per cine frame Dice Scores between models [c] and [d] (Table 3).

Following the segmentation outputs from the model, we performed CMR MVM velocity analysis for each model in the framework, where we considered the segmentation model and the post-processing stage as one segmentation framework. We excluded four cine slices whose myocardial velocities could not be generated from their segmentation from any of the models by the myocardial velocity analysis tool in the below comparison study. We firstly conducted ICC tests (Supplementary Table ST1) for each of these peak myocardial velocity values (PD, PA and PAS for the longitudinal velocity; PD, PA and PAS for the longitudinal velocity; and C1, C2 and C3 for the circumferential velocity, as specified in Section 2.6.2) generated from segmentation results. We then classified all ICCs per method specified in Section 2.6.2 (Table 4). We were able to observe that all ICCs of global values and the majority of the ICCs of regional values are classified as excellent. Additionally, we also made Bland–Altman plots for each of these peak myocardial velocity values (PD, PA and PAS for the longitudinal velocity; PD, PA and PAS for the longitudinal velocity; and C1, C2 and C3 for the circumferential velocity, as specified in Section 2.6.2) generated from different models (Supplementary Figure SF1).

### 3.2. Independent Testing

**Figure 4:**
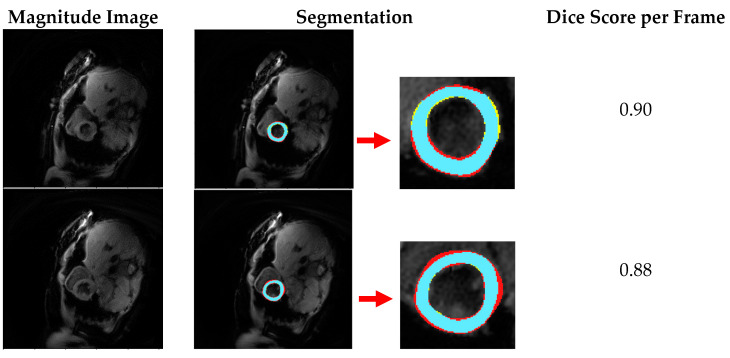
Segmentation results for sample cine frames from a subject (blue: true positive automated segmentation; yellow: false positive automated segmentation; red: false negative automated segmentation).

**Figure 5:**
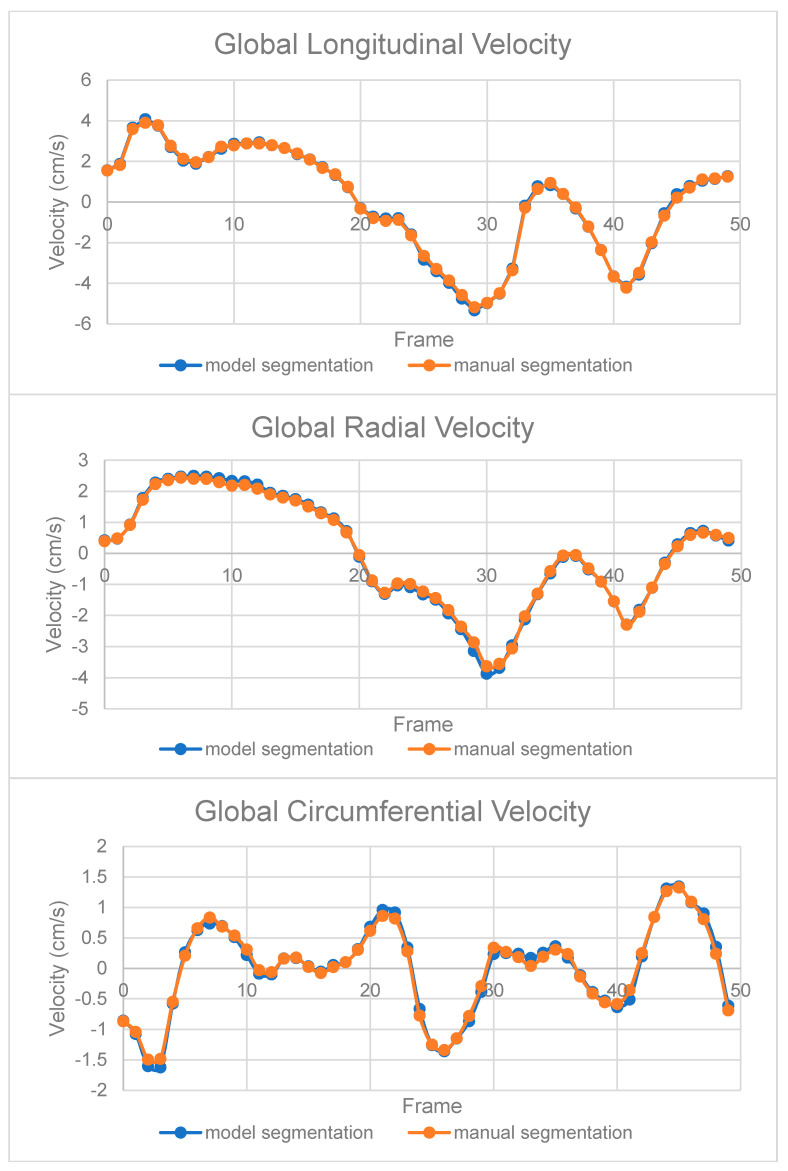
Global velocity curves of the longitudinal, radial and circumferential myocardium velocities per cine slice for a sample cine slice from a subject generated from the manual segmentation and our automated segmentation system.

**Figure 6:**
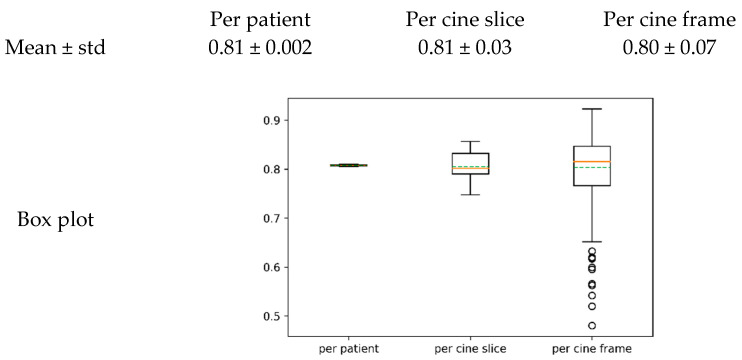
Dice Score boxplots for the independent testing datasets. In the boxplot, the orange line indicates the median value, the green line indicates the mean value and the dots around indicate the outliers. Outliers here are defined as data points above Q3 + 1.5 × IQR or below Q1 − 1.5 × IQR of the distribution. (Q1: first quartile, Q3: third quartile, IQR: interquartile range (Q3–Q1).)

In addition, the trained model with the highest Dice Score (model [d]) also performed well on further two independent testing datasets, which were not included in the training and cross-validation process (Figure 4 and Figure 5) with high Dice Score distributions (Figure 6). 

The independent testing by these two datasets further confirms the performance of our model [d] in addition to the cross-validation.

### 3.3. Inter-Observer Study

With the segmentation from the second observer (as specified in Section 2.7), we can calculate the Dice Scores between the first and the second observers as specified in Section 2.6.1, who are both very experienced CMR clinicians/physicists, as specified in Section 2.1 and Section 2.7. This provides a reference for inter-observer manual variation of the myocardium segmentation in terms of the Dice Scores. 

We then extracted the automated segmentation of the corresponding subjects by model [d] (highest performing as per Section 3.1) from Section 3.1 and calculated its distribution of Dice Scores against the first observer as per Section 2.6.1. 

**Figure 7:**
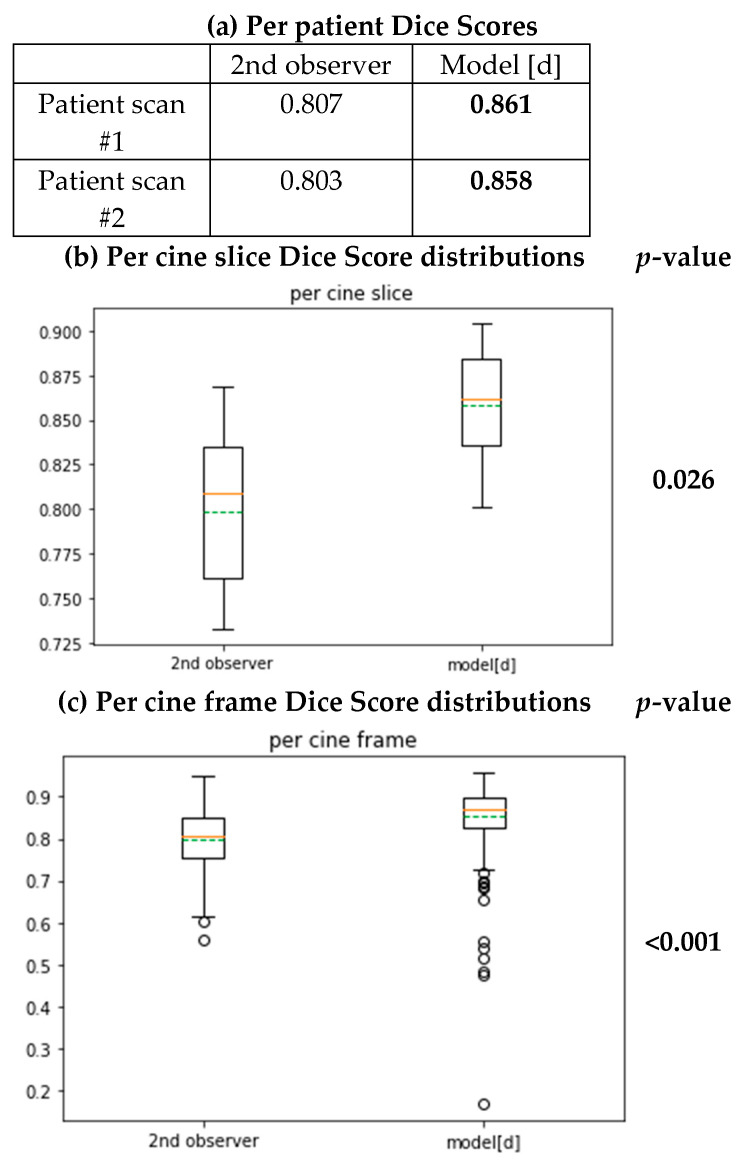
Boxplots for the Dice distribution of the 2nd observer and model [d] and its statistical tests for difference. In the boxplot, the orange line indicates the median value, the green line indicates the mean value and the dots around indicate the outliers. Outliers here are defined as data points above Q3 + 1.5 × IQR or below Q1 − 1.5 × IQR of the distribution. (Q1: first quartile, Q3: third quartile, IQR: interquartile range (Q3–Q1).)

We were then able to observe a significantly higher distribution of Dice Scores per subject and per cine slice in the automated segmentation from their box plots of Dice Scores (Figure 7). Although in the box plot for per cine frame Dice Scores, we have a few outliers that fall below the distribution of the second observer, it did achieve statistical significance by Wilcoxon signed-rank tests against the distribution obtained from the second observer (Figure 7).

### 3.4. Processing Time

By testing all models on an NVIDIA TESLA V100 GPU or using the option without the GPU as per Section 2.3, we were able to measure their processing time, respectively (Table 5). Within the processing time, the process of data loading and model loading solely takes less than 6 seconds for each of the models regardless of the employment of the GPU. We can conclude that all models are able to load and infer on a cine slice within 10.5 seconds on average using an NVIDIA TESLA V100 GPU as per Section 2.3. Even without the GPU, all models are able to complete segmentation of a single cine slice within 60.7 seconds on average. Thus, it is also worth noting that even without the GPU, the model is still able to infer each cine slice within a minute or slightly over a minute, as per the methodology mentioned in Section 2.4. This implied that a typical modern computer can also perform the automated segmentation within an acceptable period of time once the model is trained. 

Its efficiency in segmentation, together with its efficacy and accuracy compared to the currently highly tedious and time-consuming manual segmentation processes, makes the developed framework welcomed for use by clinicians.

## 4. Discussion

Our current work has served as a proof-of-concept study to introduce deep learning networks into the segmentation of left ventricles in 3Dir MVM CMR data. 

Section 3.1 and Section 3.2 have provided evidence from both methodologies of cross-valuation and independent testing to evaluate the performance of the models. The results from Section 3.1 and Section 3.2 have provided statistically strong evidence that the multi-channel can outperform the previously existing single-channel segmentation models. They have also proved that the integration of different channels (magnitudes and velocities) can provide more information and thus better performance for the automated segmentation framework. Our experiments have also provided evidence for the usefulness of these attention modules when integrating and processing multi-channel data.

Additionally, we performed myocardial velocity analysis, where we analysed the segmentation results in terms of the time derivatives of temporal physiological movements of the segmented myocardium, so we can see how similar the automatically segmented myocardium to the manually segmented myocardium when pumping blood across time frames across all three axes during an R-R interval in the ECG. From the very low bias (close to 0) and small limits of agreements in velocity curves and all peak values derived across all the longitudinal, radial and circumferential directions, we were then able to prove the efficacy and accuracy of our automated segmentation by the velocities derived from simulated temporal LV myocardium movements in addition to the Dice Scores evaluating the overlaps in the automated segmentation results.

Although we were not able to compare the segmentation results on all slices by myocardial velocity analysis as our myocardium velocity analysis tool has a very strict temporal and shape restriction on the segmentation result input, we could still analyse the majority of the cine slices and provided promising results as supporting evidence to our proposed method. We also believe that with the advancement of the algorithm with temporal and shape constraints on its output, we can mitigate this problem in future versions of this segmentation framework. From the inter-observer study, we are now able to observe the variability among human observers and have provided a reference for future technicians regarding the variation they should expect in the ground truths from different observers. The results have also provided strong evidence to suggest lower variations of our model [d] against its training dataset (or the first observer) than the second observer’s and have further confirmed evidence regarding the existence of minor inter-observer variation in manual segmentation, which can be compensated and rectified by our automated segmentation framework. However, the inter-observer study can be applied to a greater number of subjects to supplement the generality of this study.

At the current stage, our proof-of-concept study has assessed the performance of the proposed deep neural networks on healthy subjects only. We expect that a more diverse group of subjects will be introduced in further development, where 3Dir MVM CMR images will be collected from patients with cardiac disease (e.g., dilated cardiomyopathy) and further analysis will be conducted. With a more diverse group of subjects, we will be able to critically evaluate and report the benefits and shortcomings of this methodology. Additionally, with the introduction of a more diverse range of subjects, we may need to adjust and re-design the post-processing stages, as the current design of the post-processing workflow is based on the assumption that all subjects are healthy and thus that the shapes of their LV myocardium should be ellipse-like. This may be considered as a limitation of our current work and may require another layer of post-processing to mitigate any “broken” segmentations. In this paper, we were able to assess the performance of models in our very unique data with a limited number of subjects. However, more experiments on multi-centre and multi-scanner datasets with a greater number of subjects may be needed to assess the generality and reproducibility of the proposed model design. Moreover, to facilitate a larger clinical trial, more comprehensive inter- and intra-observer study will be carried out that will involve manual labelling for the clustering of clinicians with various experience. 

Another limitation of our work is that as the results show in Table 5, we have similar inference time per slice, and the inference times are model [a] ~ = model [b] ~ = model [c] < model [d]. It is of note that although our model [d] outperformed model [a] and model [b] with statistical significance, variances of model performance were small and model [d] also required more computing time.

For the design of the model network, we have noticed that there are plenty of directions for further developments. This can include the incorporation of Long Short-Term Networks (LSTM) and/or Recursive Neural Networks (RNN) into our framework to process the temporal information of the cine slices. 

## 5. Conclusions

In this study, we proved that the standard U-Net-based structure can be used for myocardium segmentation in 3Dir MVM CMR. Additionally, we proposed a multi-channel attention block to better incorporate the multi-channel information and used that to design our novel network architecture with post-processing stages to enhance the segmentation accuracy. In order to assess the performance of the designed frameworks, we evaluated them with relation to quantitative CMR MVM velocity peaks in addition to the Dice Scores. Our experiment results demonstrated the high efficacy and efficiency of our proposed automated segmentation framework.

## Figures and Tables

**Table 1:** Demographic information of the 19 subjects (18 for training and 1 for independent testing). ECG R-R intervals were approximately +/−100ms. For 10 of the subjects, volunteers were asked to provide their weights and heights without having them actually measured.

	Age (year)	Weight (kg)	Height (m)	ECG R-R Interval (ms)	Gender
**Minimum**	25	57	1.63	780	14 Males 5 Females
**Maximum**	59	90	1.95	1500	
**Mean**	38.1	73.9	1.78	1003	
**Standard Deviation**	11.4	9.3	0.09	184	

**Table 2:** Statistics (mean and standard deviation) Dice Scores of different models prior to post-processing (Bold values indicate the best performance).

	Single-Channel Data		Multi-Channel Data	
Model Type (2.2) Dice Score Type	[a] U-Net with Magnitude Data (Dice Score Mean, Standard Deviation)	[b] U-Net with Velocity Data (Dice Score Mean, Standard Deviation)	[c] U-Net with Magnitude and Velocity Data (Dice Score Mean, Standard Deviation)	[d] Proposed MCAB (Dice Score Mean, Standard Deviation)
Per subject	0.8604, 0.0324	0.8513, 0.0292	0.8672, 0.0271	**0.8679, 0.0290**
Per cine slice	0.8615, 0.0687	0.8492, 0.0784	0.8688, 0.0551	**0.8692. 0.0489**
Per cine frame	0.8596, 0.0850	0.8455, 0.0977	0.8666, 0.0713	**0.8668, 0.0747**

**Table 3:** Wilcoxon signed-rank tests to compare models using Dice Score distribution pattern per subject (a), per cine slice (b) and per cine frame (c) of the comparison study results in five-fold cross-validations using different methods.

		(a) Per Subject	(b) Per Cine Slice	(c) Per Cine Frame
Model x	Model y	*p*-value	*p*-value	*p*-value
**[a]**	**[d]**	**0.016**	**0.004**	**<0.001**
**[b]**	**[d]**	**<0.001**	**<0.001**	**<0.001**
**[c]**	**[d]**	0.809	0.367	**<0.001**
**[a]**	**[c]**	**0.046**	0.005	**<0.001**
**[b]**	**[c]**	**0.001**	**<0.001**	**<0.001**

Bold: values exhibit statistical significance.

**Table 4:** Classification of inter-class coefficients (ICCs) of CMR MV markers across models.

				[a] U_M	[b] U_P	[c] U_MP	[d] AMU_MP
	global	excellent	>0.75	**9**	**9**	**9**	**9**
		good	0.6 < x ≤ 0.75	0	0	0	0
		fair	0.4 < x ≤ 0.6	0	0	0	0
		poor	≤0.4	0	0	0	0
AS	anteroseptal	excellent	>0.75	**9**	8	**9**	7
		good	0.6 < x ≤ 0.75	0	1	0	2
		fair	0.4 < x ≤ 0.6	0	0	0	0
		poor	≤0.4	0	0	0	0
A	anterior	excellent	>0.75	**9**	8	**9**	8
		good	0.6 < x ≤ 0.75	0	1	0	1
		fair	0.4 < x ≤ 0.6	0	0	0	0
		poor	≤0.4	0	0	0	0
AL	anterolateral	excellent	>0.75	8	8	**9**	8
		good	0.6 < x ≤ 0.75	1	1	0	1
		fair	0.4 < x ≤ 0.6	0	0	0	0
		poor	≤0.4	0	0	0	0
IL	inferolateral	excellent	>0.75	**9**	**9**	**9**	8
		good	0.6 < x ≤ 0.75	0	0	0	1
		fair	0.4 < x ≤ 0.6	0	0	0	0
		poor	≤0.4	0	0	0	0
I	inferior	excellent	>0.75	**9**	8	**9**	**9**
		good	0.6 < x ≤ 0.75	0	1	0	0
		fair	0.4 < x ≤ 0.6	0	0	0	0
		poor	≤0.4	0	0	0	0
IS	inferoseptal	excellent	>0.75	**9**	**9**	**9**	**9**
		good	0.6 < x ≤ 0.75	0	0	0	0
		fair	0.4 < x ≤ 0.6	0	0	0	0
		poor	≤0.4	0	0	0	0

**Table 5:** Processing time of different models with or without the GPU. The time here is inclusive of data loading, model loading and inference.

Processing Time (s)/#Cine Slices Tested (Mean Time Per Cine Slice (s))	Model [a]	Model [b]	Model [c]	Model [d]
With GPU	21.1/3 (7.03)	21.8/3 (7.27)	21.5/3 (7.17)	31.4/3 (10.5)
Without GPU	96.1/3 (32.0)	96.1/3 (32.0)	96.4/3 (32.1)	182/3 (60.7)

## Supplementary Materials

The following are available online at https://www.mdpi.com/2075-4418/11/2/346/s1, Supplementary Table ST1—Inter-class coefficients (ICCs) of CMR MVM markers across the models (ST1.xlsx). Supplementary Figure SF1—Bland–Altman figures (SF1.png)
