# Supplementary figures and images for: Fast and Automated Segmentation for the Three-Directional Multi-Slice Cine Myocardial Velocity Mapping
Source: Diagnostics (Basel). 2021 Feb 19;11(2):346. doi: 10.3390/diagnostics11020346 (PMC7922945; doi:10.3390/diagnostics11020346)

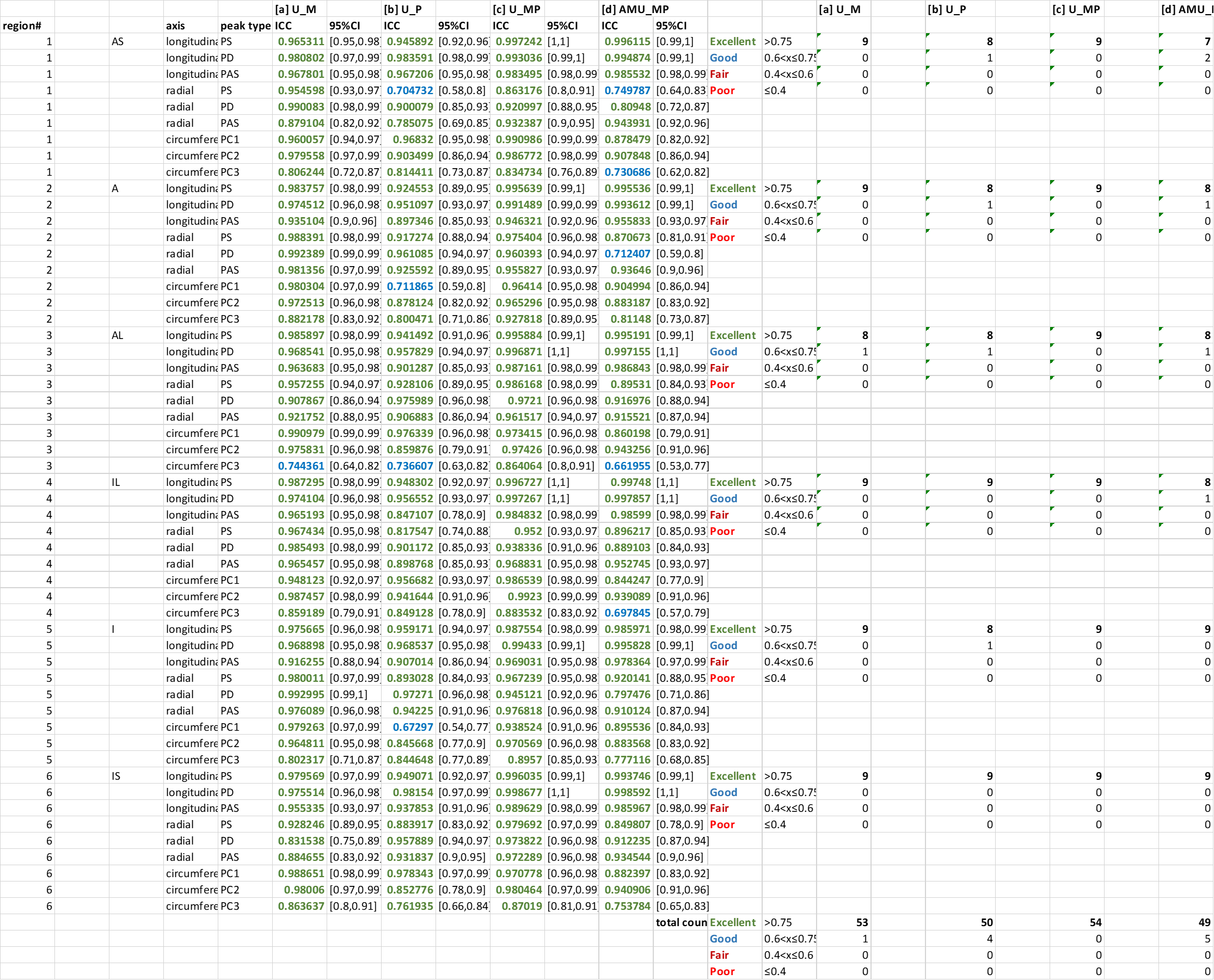

Supplement: Supplementary file 1 [file diagnostics-11-00346-s001.zip › 1.png]

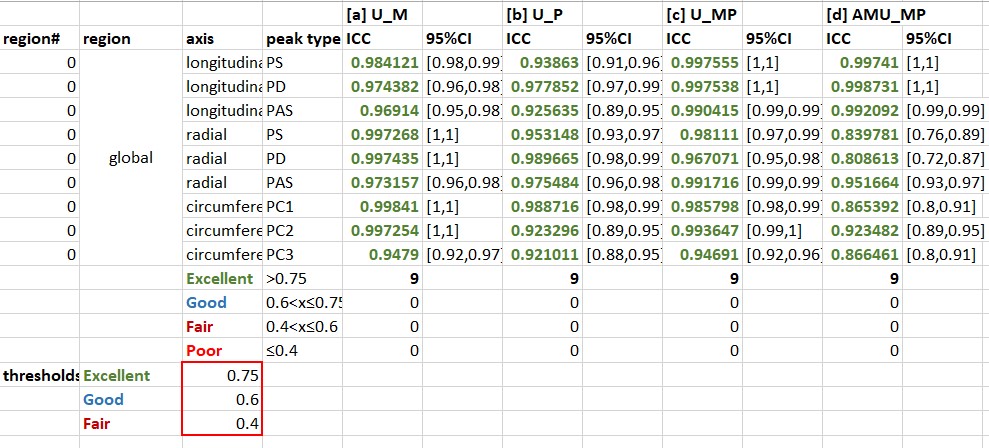

Supplement: Supplementary file 1 [file diagnostics-11-00346-s001.zip › 2.jpg]
